# Mitigating Diarrhoea-Related Inflammation in Frail Older Adults with Postbiotic-Enhanced Oral Rehydration Solution: Insights from a Randomised, Double-Blind, Placebo-Controlled Study

**DOI:** 10.3390/geriatrics10020034

**Published:** 2025-03-01

**Authors:** Julian Andrés Mateus Rodríguez, Patricia Rodríguez Sanz, Edgar Kostandyan, Rubén Palacios Sanchez, María Luz Pino Roque, Patricia Chaves Vasquez, Pedro Roy Millán

**Affiliations:** 1 Hospital d’Atenció Intermedia Colisée Barcelona Isabel Roig, 08030 Barcelona, Spain; prodriguez@colisee.es (P.R.S.); rpalacios@colisee.es (R.P.S.); mlpino@colisee.es (M.L.P.R.); 2 Hospital Mare de Déu de la Mercè, Hermanas Hospitalarias, 08042 Barcelona, Spain; ekostandyan.merced@hospitalarias.es (E.K.); pchavesv.merced@hospitalarias.es (P.C.V.); proym.merced@hospitalarias.es (P.R.M.); 3 Facultat d’Infermeria, Universitat de Barcelona, 08907 Barcelona, Spain

**Keywords:** postbiotics, ABB C22, *Saccharomyces boulardii*, diarrhoea, gastrointestinal inflammation, oral rehydration salts

## Abstract

**Background/Objectives:** Diarrhoea in older adults can lead to dehydration and malnutrition, impaired gut barrier function, and reduced quality of life. Unresolved inflammation during diarrhoea episodes contributes to relapse and complications. This randomised study evaluated the effects of a novel oral rehydration solution (ORS) with the postbiotic ABB C22^®^, known for its anti-inflammatory properties, on diarrhoea-associated inflammation in an elderly population. **Methods**: A randomised, double-blind, placebo-controlled, parallel-group trial was conducted at two hospital centres in Barcelona, Spain. Forty-seven participants aged ≥65 years with diarrhoea (*n* = 47) were randomised (1:1) to receive either ABB C22^®^-enriched ORS or placebo ORS for up to 14 days. Randomization was stratified by centre using a computer-generated sequence. Participants, caregivers, and outcome assessors were blinded. Primary endpoints were changes in faecal inflammatory biomarkers (calprotectin and lactoferrin) and blood immunoglobulin A. Secondary endpoints included changes in stool consistency (Bristol Stool Scale) and treatment tolerability. **Results**: Of the 47 participants, 42 completed the trial (21 per group). At day 14, the ORS + ABB C22^®^ group showed greater reductions in faecal calprotectin and lactoferrin levels compared to the placebo group. Lactoferrin-positive cases were halved by day 3 in the intervention group. Stool consistency improved in both groups. No adverse events were reported in either group. **Conclusions**: ABB C22^®^-enriched ORS exhibited superior anti-inflammatory effects compared to standard ORS while achieving similar improvements in stool consistency. These findings suggest that postbiotic-enriched formulations represent a promising approach to better address the management of diarrhoea which is often accompanied by gut inflammation. The study protocol was registered in ClinicalTrials.gov (NCT06738420; date: 16 December 2024).

## 1. Introduction

Acute gastroenteritis is an inflammation of the gastrointestinal system lining, particularly stomach and intestines, of which the main symptoms usually include vomiting and diarrhoea. Diarrhoea is a common illness in older adults that can lead to dehydration and malnutrition, impair gut barrier function, and negatively impact quality of life [1]. It is defined by the World Health Organization (WHO) as having three or more loose or liquid stools per day or having more stools than is normal for that person [2]. Acute diarrhoea is defined as an abnormally frequent discharge of semisolid or fluid faecal matter, lasting less than 14 days [2]. It is mainly caused by infections, with *Clostridium difficile* being the most common in older adults, followed by *E. coli*, rotavirus, and other pathogens of different origin, including bacteria, fungi, or parasites [3,4,5]. Chronic diarrhoea, in contrast, lasts for more than 2–4 weeks and is usually associated with underlying disease or secondary to medications, which are especially common in the elderly [6,7]. Antibiotic-associated diarrhoea occurs when treatment with antibiotics affects the beneficial microbiota, decreasing beneficial bacteria and allowing for an overgrowth of pathogens and dysbiosis [8]. Enteral nutrition therapy (tube feeding) is also commonly associated with diarrhoea. Although preferred over parenteral nutrition (intravenous feeding), enteral nutrition may need to be discontinued if diarrhoea or other gastrointestinal side effects cannot be adequately managed [9,10,11].

Diarrhoea in older adults is a significant health concern that can lead to severe complications, particularly due to the increased risk of dehydration and its impact on overall health and quality of life. Long-lasting or cyclic diarrhoeal episodes can lead to suboptimal nutrient absorption, especially for protein and essential vitamins and minerals [1]. Recurrent diarrhoeal episodes can lead to chronic intestinal inflammation, which causes reduced nutritional intake. The consequent malnutrition can in turn increase the risk of reinfection and recurrence of the episodes, and reduce resistance to new enteropathogens, creating a vicious cycle. In other words, untreated inflammation prolongs malnutrition and increases the risk of recurring diarrhoea, thus delaying recovery. The resulting impairment of gut barrier function also leads to weakened protection from pathogens, which can easily cross into the bloodstream, causing septicaemia and worsened inflammation. Diarrhoea in the elderly can also cause severe dehydration [12], leading to cardiovascular complications like fluctuations in blood pressure and electrolyte imbalance, kidney dysfunction [13], and impaired cognitive performance and motor coordination [14].

Probiotics can help manage the symptoms of diarrhoea [15]. *Saccharomyces boulardii* is the most studied yeast strain with probiotic properties for human health and nutrition [16]. Together with *Lactobacillus rhamnosus GG*, it is one of the only two yeast probiotic strains with level 1 evidence in the World Gastroenterology Organization (WGO) probiotics and prebiotics guidelines [17]. *S. boulardii* has been tested in clinical studies for many gastrointestinal health conditions [18] including acute [19] or antibiotic-associated diarrhoea [16,20], *C. difficile* infection [16,21,22], irritable bowel syndrome [16,23], inflammatory bowel disease [16,24], and immune disorders [16]. Its protective gastrointestinal effects are driven, among other mechanisms of action, by its superior capacity to modulate the gut microbiota and promote the production of short-chain fatty acids (SCFAs) [8]. SCFAs, particularly butyric acid, contribute to the active transport of ions in the colonic epithelium, promoting water reabsorption and consequently reducing its excretion [25]. This effect has been proven clinically, showing that *S. boulardii* induces the production of SCFAs and provides a protective effect on diarrhoea in patients on long-term total enteral nutrition [26]. However, many patients on enteral nutrition programmes or suffering from acute diarrhoea episodes in the hospital setting may be immunocompromised, which raises concerns for treating them with live probiotics [27,28].

Tyndallised (heat-killed) yeasts, known as postbiotics, overcome the concerns of administering live probiotics, while retaining many beneficial properties [27]. ABB C22^®^ is a combination of three tyndallised postbiotic yeasts, *S. boulardii* ABB S3, *Saccharomyces cerevisiae* ABB S6, and *Kluyveromyces marxianus* ABB S8. ABB C22^®^ protects the integrity and function of enterocytes by different and complementary mechanisms that include enhancing gut barrier function, inducing a strong anti-inflammatory effect, protecting against rotavirus infection, and modulating microbiome properties [29]. ABB C22^®^ also provides highly bioavailable zinc, an essential micronutrient recommended by the WHO for managing diarrhoea by reducing the duration and severity of episodes [30]. The yeasts in ABB C22^®^ retain their cellular structure after tyndallisation, thus preserving many of their beneficial mechanisms of action, and supporting their use in a wide range of patients with gastrointestinal conditions [31,32,33]. Furthermore, the stability of these bioactive postbiotic yeasts, and the absence of drug interactions makes them suitable for direct incorporation into enteral nutrition formulations or oral rehydration solutions (ORSs), facilitating their use in patients who are already taking multiple medications (including antibiotics), or who have difficulty eating solid food. In this pilot study, we assessed the benefits of dietary supplementation with new medical nutrition formulas containing postbiotics with effects in protecting gastrointestinal health. Specifically, we tested an ORS enriched with ABB C22^®^ yeast postbiotic in older adults with diarrhoea and signs of dehydration, and measured its effect on inflammatory biomarkers and faecal consistency.

## 2. Materials and Methods

### 2.1. Study Design

This study was a multi-centre, randomised, double-blind, placebo-controlled, parallel-group trial conducted at Hospital d’Atenció Intermedia Colisée Barcelona Isabel Roig and Hospital Mare de Déu de la Mercè, which is part of the FIDMAG Hermanas Hospitalarias Research Foundation in Barcelona, Spain. The objective of the study was to evaluate the benefits of dietary supplementation with new medical nutrition formulas containing postbiotics with gastrointestinal protective effects (with level 1 evidence in the WGO probiotics and prebiotics guidelines), in patients with diarrhoea and gastrointestinal symptoms, compared to the study sites’ standard of care [34]. Specifically, an oral rehydration solution (ORS) enriched with the ABB C22^®^ yeast postbiotic was tested in older adults with diarrhoea and signs of dehydration. During the study, participants received either active product (ORS + ABB C22^®^) or standard treatment (ORS + placebo). The primary outcomes were changes in the faecal inflammatory markers calprotectin and lactoferrin; and serum immunoglobulin A (IgA) at the end of the supplementation period compared to baseline. The secondary outcomes included measuring the reduction in the duration and severity of diarrhoea (number of depositions), gastrointestinal symptoms (such as diarrhoea, vomiting, and regurgitation), and changes in faecal consistency (Bristol Stool Scale). Change in serum levels of C-reactive protein was included as an exploratory parameter. The trial was conducted from March 2023 to March 2024. The study protocol received approval from the Clinical Research Ethics Committee of Fundació Unió Catalana d’Hospitals (code CEI 22/92; approval date 23 November 2022) and the FIDMAG Hermanas Hospitalarias Research Foundation (code PR-2022-27; approval date 26 January 2023), and was registered in ClinicalTrials.gov (NCT06738420). All procedures adhered to the Declaration of Helsinki, and participants provided written, informed consent. Participant records and information were anonymized.

### 2.2. Study Population Selection and Randomization

This trial was planned as a pilot study in a real-life population in a controlled environment where healthcare and diet were guaranteed and monitored, unlike many interventional studies in these disorders. The study was planned as a pilot study because there are no reliable data on postbiotics in patients with diarrhoea with altered faecal calprotectin or lactoferrin levels that could help establish the required sample size. Initially, a total recruitment of 50 patients with diarrhoea was planned, with 25 being assigned to the ORS + ABB C22^®^ group, and 25 to the ORS + placebo group. The recruitment capacity of the centres and a drop-out rate of 10% was considered to determine the sample size, considering that they are well-controlled hospitalised patients. Patients who were admitted to long-stay centres at Hospital d’Atenció Intermedia Colisée Barcelona Isabel Roig and Hospital Mare de Déu de la Mercè who had been diagnosed with diarrhoea could participate in the study. Diarrhoea was considered as an increase in daily faecal weight of >200 g as per protocol of the study sites. For inclusion in the study, participants had to be diagnosed with diarrhoea, had to require oral rehydration therapy according to the site protocol, had to be able to take the study product orally, and had to understand the study procedures. Exclusion criteria were as follows: history of allergy; idiosyncratic hypersensitivity or adverse reactions to the active principle or to any of the excipients; history or evidence of any medical conditions or medication used that, in the opinion of the principal investigator, could affect the safety of the subjects or interfere with the study evaluations; end-of-life patients; and patients under amitriptyline (anticholinergic/antidepressant) and mesalazine treatment, under antibiotic treatment, or taking laxatives. Upon enrolment, participants were randomly assigned in a 1:1 ratio to receive either ORS + ABB C22^®^ or ORS + placebo. The randomization sequences were generated using the R statistical package function sample, and were generated separately for female and male participants, and according to age and recruitment block. The randomization schedule was provided by the study sponsor to the entity providing the central randomization and was readily available to the lead investigator in case of emergency. Participants, caregivers, and outcome assessors were blinded.

### 2.3. Study Treatment and Intervention

**Figure 1:**
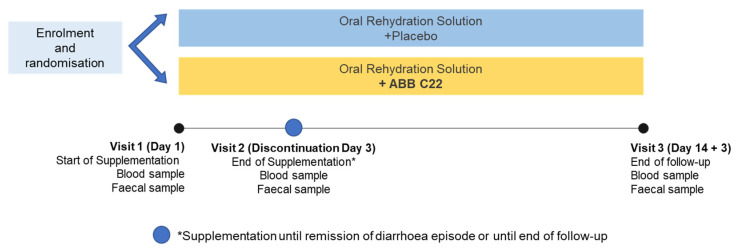
Study participants were randomised to receive either the active product of oral rehydration solution (ORS) + ABB C22^®^ or a standard treatment of ORS + placebo. Blood and faecal samples were collected for analysis during study visits at days 1, 3, and 14 (end of study).

The active product, ORS + ABB C22^®^, (ABbiotek Health, a business division of AB Mauri, Peterborough, UK), was a strawberry-flavoured ORS enriched with a postbiotic blend of *S. boulardii* ABB S3^®^, *S. cerevisiae* ABB S6^®^ (rich in zinc), and *K. marxianus* ABB S8 (ABB C22^®^). The postbiotic dose was established to ensure enough postbiotic cells for anti-inflammatory effect and, in particular, enough *Saccharomyces boulardii* cells to be in line with previous studies with this probiotic yeast [17]. The standard treatment was ORS with placebo instead of ABB C22^®^. ORS consisted of a 200 mL hypotonic beverage in ready-to-drink tetra-bricks, containing water, glucose, sodium citrate, sodium chloride, potassium chloride, natural strawberry flavour, acidity regulator, and sweetener. The formula followed the WHO guidelines for oral rehydration [30]. Participants were administered either active product (ORS + ABB C22^®^) or standard treatment (ORS + placebo) over 14 days, or until remission of the diarrhoea episode, whichever occurred sooner. The pharmacy services of Colisée Barcelona Isabel Roig and Hospital Mare de Déu de la Mercè were responsible for supplying the study treatments in labelled Tetra Brik 200 mL cartons with identical appearance. Participants were instructed to take 3 cartons/day orally, distributed with meals. Treatment assignment (ORS + ABB C22^®^ or ORS + placebo) was concealed until the end of the study. Eligibility criteria were checked, informed consent was obtained, randomization was performed, and the nutritional supplement was provided (Figure 1). Participants started the supplementation period at visit 1 (day 1). During visit 2 (day 3), participants were evaluated for their response to treatment. If the diarrhoea had stopped, then they could discontinue supplementation. A follow-up visit (visit 3) took place on day 14. At all visits, blood and faecal samples were collected, safety assessments were performed, and diarrhoea duration and severity (number of depositions) and faecal consistency (Bristol Stool Scale) were assessed. Serious or unexpected adverse events were screened during every visit, and participants were instructed to use a diary card for recording side effects.

### 2.4. Laboratory Analyses

Analyses of serum, blood, and stool samples were conducted at Synlab Barcelona (Carrer Verge de Guadalupe, 18 08950 Esplugues de Llobregat). For faecal inflammatory parameters, lactoferrin and *C. difficile* antigen and toxins were measured by immunochromatography and expressed as positive or negative. Faecal calprotectin levels were measured by chemiluminescence (Liaison, Diasorin, Saluggia, Italy) and expressed as µg/g. Serum total IgA levels and blood C-reactive protein were measured by turbidimetry (AU5800, Beckman Coulter Life Sciences, Indianapolis, IN, USA). Stool samples were cultured to identify the presence of *Salmonella*, *Shigella*, *Yersinia*, or *Campylobacter*. Blood count parameters, including red cells, haemoglobin, haematocrit, leukocytes, platelets, MCV, MHC, MCHC, RDW, and MPV, were measured by flow cytometry (DxH 800, Beckman Coulter Life Sciences). As part of the safety monitoring and evaluation of the product, serum concentrations of chloride, potassium, and sodium were determined by ion-selective electrode (AU5800, Beckman Coulter Life Sciences) and expressed as mEq/L. The levels of enzymes and metabolites in serum, including urea, creatinine, uric acid, GGT, GOT, GPT, and LDH, were measured by spectrometry (AU5800, Beckman Coulter Life Sciences). Lactate levels in serum were also determined by spectrometry (AU680, Beckman Coulter Life Sciences, Indianapolis, IN, USA).

### 2.5. Statistical Analysis

Statistical analyses were performed by an external service (2Smestres, Barcelona, Spain). All study variables were defined, and descriptive statistics were used according to the nature of the variable. Continuous variables are described as mean and standard deviation (SD) or median and interquartile range. Categorical variables are presented as absolute frequencies and percentages. A linear mixed-effects model (LMM) was employed for repeated measures using restricted maximum likelihood (REML). This model includes a random intercept for each participant, adjusts for the baseline value as a covariate, and accounts for interactions between treatments and evaluation time points (days 1, 3, and 14). The chosen covariance structure was unstructured to capture within-subject correlations. Additionally, we conducted estimated marginal means comparisons to quantify specific differences between groups over time and within groups. This approach is more robust than more traditional methods, such as paired *t*-tests or repeated-measures ANOVA, particularly in handling missing data and modelling intra-individual variability. The statistical programme R version 3.6.2 or higher for Windows was used for data treatment and analysis (R Foundation for Statistical Computing, http://www.r-project.org (accessed on 18 October 2022)).

## 3. Results

### 3.1. Participants

**Figure 2:**
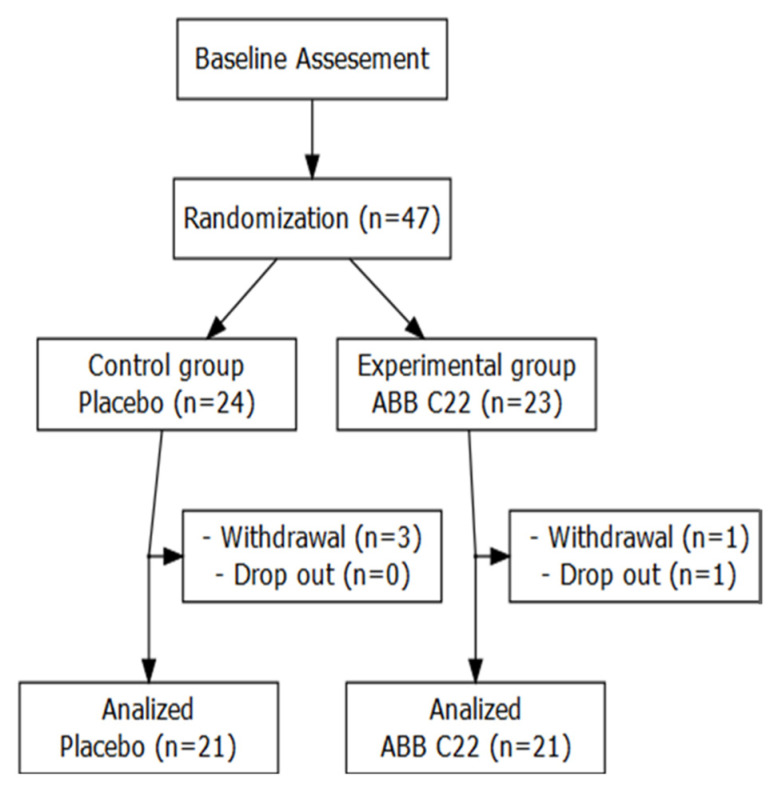
Patient flow diagram. After randomization, three patients in the ORS + placebo group were withdrawn from the study due to the deterioration of their baseline health status, which required referral to an acute care hospital. One patient in the ORS + ABB C22^®^ group was removed from the analysis because they did not take the study treatment, and another patient dropped out of the study because they did not wish to continue.

Forty-seven participants were enrolled in the study and randomly assigned to either the ORS + ABB C22^®^ group (*n* = 23) or ORS + placebo group (*n* = 24). Final analyses were conducted with 42 participants ranging in age from 57 to 101 years (32 females), with 21 participants in each group (Figure 2, Table 1). Two cases of *Clostridium difficile* infections were identified during the study, suggesting a low prevalence in the study population. Patients who tested positive for *C. difficile* were treated with vancomycin. Coproculture confirmed that there were no cases of *Salmonella*, *Shigella*, *Yersinia*, or *Campylobacter* infection in the study.

### 3.2. Biomarker Levels

#### 3.2.1. Inflammatory Markers

**Figure 3:**
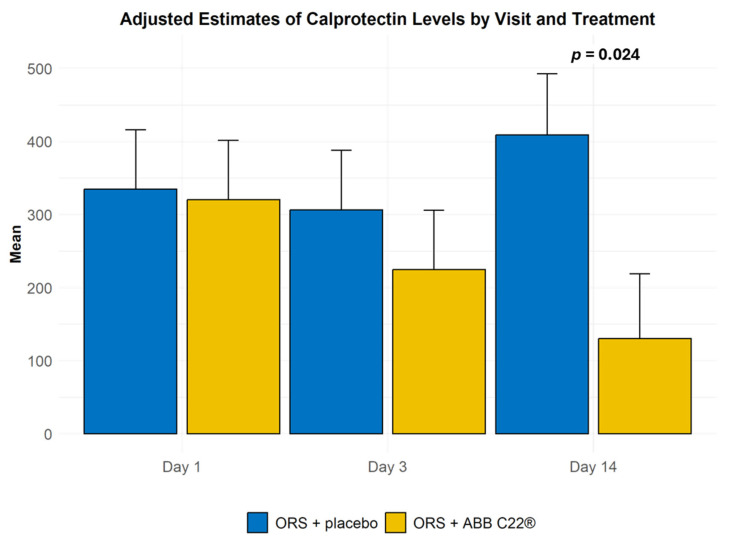
Faecal calprotectin levels in the ORS + ABB C22^®^ group were decreased already after 3 days of treatment. Levels continued to decrease until day 14, when they showed a clear and statistically significant difference from the ORS + placebo group.

The primary outcome of the study was changes in the faecal levels of inflammatory markers from baseline to the end of the study. In the ORS + ABB C22^®^ group, stool concentrations of calprotectin decreased after just 3 days of treatment from a mean of 310 µg/g at day 1 to 208 µg/g at day 3, whereas in the ORS + placebo group, this parameter decreased from 337 µg/g at day 1 to 308 µg/g at day 3 (Table 2 and Figure 3). Calprotectin levels in the ORS + ABB C22^®^ group improved steadily over time and became clear and statistically significant at day 14. Variations within the same group at different timepoints were not statistically significant. For faecal lactoferrin, the number of participants who tested positive decreased in the ORS + ABB C22^®^ group from five (25%) at day 1 to three (14.3%) at day 3, while the ORS + placebo group showed no changes after 3 days of treatment (Table 2). At day 14, the number of lactoferrin-positive patients decreased to two (11.1%) in the ORS + ABB C22^®^ group compared with four (21.1%) in the ORS + placebo group. Serum IgA levels in the ORS + ABB C22^®^ group decreased at day 3 but recovered to near-baseline levels by day 14. In contrast, the ORS + placebo group showed a tendency towards decreasing IgA levels across all three visits. Changes in serum concentrations of IgA (Table 2) and serum levels of C-reactive protein (Table S1) were not statistically significant.

#### 3.2.2. Faecal Consistency (Bristol Stool Scale)

**Figure 4:**
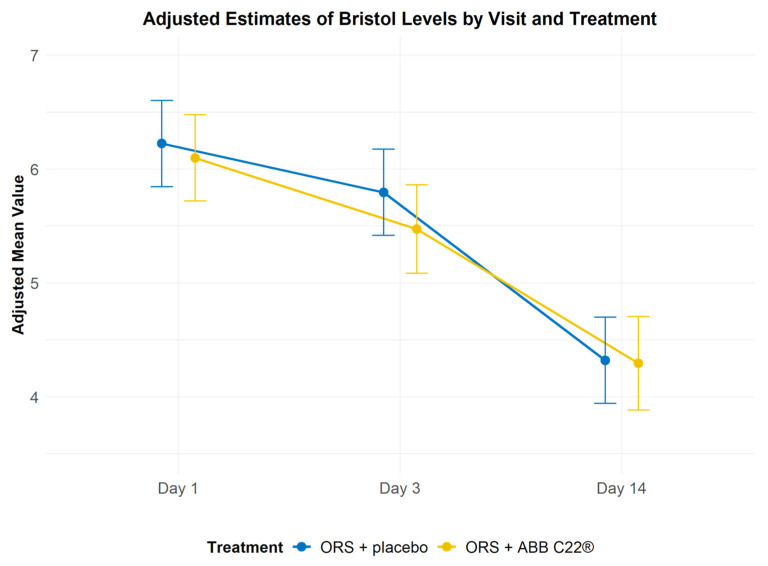
Bristol Stool Scale scores recorded at study visits. In both the ORS + ABB C22^®^ group and the ORS + placebo group, stool shape and consistency decreased from type 7 to type 4 over the course of the study.

Bristol Stool Scale scores decreased over time at similar rates in both treatment groups, and differences between the groups were not statistically significant (Table 3 and Figure 4). Both the ORS + ABB C22^®^ and the ORS + placebo group showed statistically significant decreases (improvements) in faecal consistency from day 1 to day 14 (Table 4).

### 3.3. Tolerability and Safety

The active study product was well tolerated. Nausea and very sweet flavour were reported by two participants assigned to treatment with ORS + ABB C22^®^. Both episodes were uneventful. No serious adverse events were reported. Blood analyses conducted to evaluate safety revealed no significant changes during or after supplementation. In the ORS + placebo group, three participants were referred to an acute care hospital due to the deterioration of their underlying disease, which was not related to study treatment.

## 4. Discussion

Our study assessed the effects of ORS enriched with the postbiotic ABB C22^®^ on diarrhoea and associated inflammation in older adults. Participants who were treated with ORS + ABB C22^®^ showed a decrease in faecal levels of calprotectin after just three days of treatment, and levels continued to decrease until the end of the study at day 14. Elevated faecal calprotectin levels are a known good predictor of gut inflammation [35]. Found primarily in the cytoplasm of neutrophils, calprotectin is a biomarker of neutrophil influx, which is elevated in several inflammatory conditions [36,37]. Faecal calprotectin has good stability in faeces and shows direct correlation with the degree of intestinal inflammation [38]. It has therefore become a non-invasive, reliable, and cost-effective marker for quantifying mucosal inflammation and is widely used in the diagnosis and monitoring of gastroenteritis in adults [39,40] and children [41,42,43], and of inflammatory bowel disease [44]. At day 14 of our study, participants who received ORS + ABB C22^®^ showed a significant reduction in faecal calprotectin levels, showing a mean value of 135 µg/g compared with 418 μg/g in those who received ORS + placebo. For the diagnostic use of faecal calprotectin, a reference limit of 150 µg/g has been proposed to indicate active disease [37]. In the context of this threshold, treatment with ORS + ABB C22^®^ led to the normalisation of faecal calprotectin levels to below those of active disease in only 3 days. It is important to note that several factors contribute to variability in faecal calprotectin threshold values, including differences in study populations [37,45]. In our study, participants with any type of diarrhoea could be included, so there was variability in the underlying cause and severity of symptoms. Furthermore, other conditions such as neoplastic disease, diverticulitis, Crohn’s disease, and food allergies are also associated with increased calprotectin levels as intestinal inflammation is common to them all. Increased calprotectin levels have also been associated with some prescription medications in chronic patients, like omeprazole, and analgesics, such as diclofenac [46]. Moreover, participants in our study were older adults who had been admitted to hospital, and many of them were receiving multiple medications.

Faecal lactoferrin represents another inflammatory biomarker with recognised value in infectious gastroenteritis [43,47]. For faecal lactoferrin, the number of participants who tested positive in the ORS + ABB C22^®^ group decreased to nearly half by day 3 of treatment (five to three participants), while there was no change in the ORS + placebo group, with nine participants testing positive at both day 1 and day 3. There were too few participants in the study who tested positive for faecal lactoferrin for the result to reach statistical significance. Nevertheless, the decrease found in the ORS + ABB C22^®^ group was notable.

Standard oral rehydration solutions aim to treat diarrhoea by rebalancing electrolytes and water, and do not act on intestinal inflammation. The reduction in faecal calprotectin and lactoferrin levels in participants who received ORS + ABB C22^®^ indicates that this combination of postbiotic yeasts improves diarrhoea-associated inflammation. We have previously shown that ABB C22^®^ protects the integrity and function of enterocytes by multiple complementary mechanisms, such as strengthening gut barrier function and inducing a strong anti-inflammatory effect [29]. These effects are similar to those seen with probiotics, which can stimulate the immune system, compete for intestinal epithelial cell binding sites, and modulate the gut microbiome [48]. Their use primarily in children with acute diarrhoea is associated with reduced severity and duration of illness (an average of one day less of illness).

Changes from baseline to the end of the study for serum concentrations of IgA and C-reactive protein were not statistically significant. IgA and C-reactive protein are important biomarkers in the evaluation of inflammatory processes; however, they are less specific compared with faecal calprotectin and lactoferrin, which correlate to intestinal inflammation. Mean IgA levels at baseline were within the normal range, and thus big changes were not expected. C-reactive protein does not usually increase in acute diarrhoea, and it was therefore monitored to identify patients who could be affected by systemic inflammation due to other causes. The lack of abnormal baseline results and no significant variation in this biomarker during the study support the assumption that gut inflammation signalled by calprotectin and lactoferrin was due to the acute diarrhoea episode.

Treatment with ORS + ABB C22^®^ showed clinical improvements in participants compared with ORS + placebo. Faecal consistency reported with Bristol Stool Scale scores improved in both treatment groups. This result was expected because all participants received oral rehydration, which is the current standard of care for diarrhoea. Because standard oral rehydration solutions work by restoring electrolyte balance, they are especially effective in improving faecal consistency. Within each treatment group, there was a statistically significant improvement in Bristol scores from day 1 to day 14, and from day 3 to 14, highlighting the self-limiting nature of diarrhoea and the effects of oral rehydration. From day 1 to day 3, the score difference was almost significant in participants who received ORS + ABB C22^®^ (*p* = 0.0631), compared with those who received ORS + placebo (*p* = 0.2562), indicating that ABB C22^®^ may help to improve faecal consistency sooner than standard ORS treatment.

Two crucial factors that play a decisive role in managing diarrhoea are dehydration and episode recurrence. When it comes to managing diarrhoea in older adults, there are several strategies to tackle dehydration that can help alleviate symptoms and promote recovery. These include staying hydrated, implementing dietary changes, and considering over-the-counter medications and, in some cases, prescription medications. To manage the recurrence of diarrhoeal episodes, restarting normal ingestion of foods is critical [49]. Eating foods that are low in fibre may aid in making stools firmer. A bland ‘BRAT’ diet, including bananas, toast, oatmeal, white rice, applesauce, and soup or broth, is well tolerated and may improve symptoms [27]. Anti-diarrheal therapy with anti-secretory or anti-motility agents may be started to reduce the frequency of stools. However, anti-motility drugs like loperamide can lead to post-treatment constipation and have been associated with intestinal complications such as toxic dilatation of the colon or prolonged illness when used in bacterial inflammatory diarrhoea [50,51]. Furthermore, their use should be avoided in patients with bloody diarrhoea or high fever because they can worsen severe intestinal infections. Empiric antibiotic therapy with oral fluoroquinolone can be considered in patients with more severe symptoms but may also lead to adverse effects such as dysbiosis or antibiotic-associated diarrhoea. While these solutions can provide relief from symptoms, none of them address the inflammatory component of the diarrheal episode.

Diarrhoea can negatively impact the nutritional status of the individual through reduced food intake and worsened gut nutrient absorption, which can consequently impair gut barrier protection and the readiness of the immune system, increasing the risk of new infections. This cascade of events loops into a vicious cycle where malnutrition and diarrhoea risk tend to worsen one another because of the gut-associated inflammation, which alters gut mucosal function and permeability. The control of this inflammatory process is key to support fast and complete recovery from diarrhoea.

All participants in our study received the standard of care with oral rehydration solution to replenish fluid and electrolyte loss [52]. The addition of ABB C22^®^ supplementation showed an effect on inflammatory biomarkers, confirming its mechanism of action as a useful agent to stop diarrhoea-associated inflammation in the gut. In addition, ABB C22^®^ is a source of highly bioavailable zinc, a well-established micronutrient for managing diarrhoea and preventing new episodes [53]. No serious adverse events related to treatment with ABB C22^®^ were reported. The effect of ORS + ABB C22^®^ on reducing gastrointestinal inflammation highlights its potential as a new strategy for treating diarrhoea without the adverse effects associated with other pharmacological treatments.

### Limitations and Future Directions

A notable limitation of our study is the absence of microbiome analysis, which could have provided valuable insights into the relationship between intestinal flora and recovery from diarrhoea. The study was further limited by the small sample size (*n* = 47), particularly for the number of participants who tested positive for faecal lactoferrin. The variability in the study population, which included poly-medicated older adults with diarrhoea of various underlying causes, may limit the generalizability of our findings. Nevertheless, the observed effects of ABB C22^®^ treatment on diarrhoea-associated inflammation remain clinically relevant and provide evidence for its application in diarrhoea management. Future studies should aim to follow patients for a longer period to better understand the long-term benefits of ABB C22^®^ in the recovery of gastrointestinal function after a diarrhoeal episode.

## 5. Conclusions

Diarrhoea-associated inflammation has a strong impact on disease outcome, becoming a risk factor for dehydration and episode recurrence. However, it is usually neglected during treatment. Current treatments like ORS target water and electrolyte loss, thus improving stool consistency, but are unable to counteract inflammation. The addition of postbiotic ABB C22^®^ to the standard of care with ORS leads to decreases in faecal biomarkers of gastrointestinal inflammation in older adults with diarrhoea. Furthermore, ABB C22^®^ provides highly bioavailable zinc, which helps to improve nutritional status in people with diarrhoea and lessens the severity of episodes. ABB C22^®^ thus offers a novel approach to managing diarrhoea by improving the associated gastrointestinal inflammation, which current solutions do not address.

## Figures and Tables

**Table 1:** Age and sex of study participants at baseline.

	ORS + Placebo	ORS + ABB C22^®^
	*n* = 21	*n* = 21
Age, mean (SD) years	78.4 (8.79)	83.5 (9.46)
Sex, *n* (%):		
Male	5 (23.8%)	5 (23.8%)
Female	16 (76.2%)	16 (76.2%)
Aetiology:		
Infectious (*C. difficile*)	2 (9.5%)	0 (0%)
Non-infectious	19 (80.5%)	21 (100%)

**Table 2:** Faecal and serum inflammatory biomarkers at study visits.

Biomarker and Study Visit		ORS + Placebo	ORS + ABB C22^®^	
	*n*	*n* = 21	*n* = 21	*p*-Value
Faecal calprotectin: Mean (SD) µg/g				
Day 1	40	337 (496)	310 (679)	0.901
Day 3	42	308 (284)	208 (261)	0.480
Day 14	37	418 (592)	135 (129)	0.024
Faecal lactoferrin: *n* (%) with positive result				
Day 1	41	9 (42.9%)	5 (25.0%)	0.614
Day 3	42	9 (42.9%)	3 (14.3%)	0.121
Day 14	37	4 (21.1%)	2 (11.1%)	0.692
Serum IgA: Mean (SD) mg/dL				
Day 1	38	279 (150)	247 (124)	0.830
Day 3	37	283 (142)	237 (111)	0.532
Day 14	33	285 (131)	271 (128)	0.463

Faecal calprotectin (in µg/g) and serum IgA levels (in mg/dL) are shown as mean (standard deviation). Faecal lactoferrin is shown as number of subjects with positive faecal lactoferrin result (*n*) and percentage (%). The *p*-values were obtained using a mixed-effects model for repeated measures (MMRM), adjusted for baseline values. The model includes interaction terms between treatment and visits (day 1, day 3, and day 14), allowing for the evaluation of differences within each treatment and between treatments over time.

**Table 3:** Mean (SD) Bristol Stool Scale scores reported at study visits.

Study Visit	ORS + Placebo	ORS + ABB C22^®^
	*n* = 21	*n* = 21
Day 1	6.29 (0.72)	6.05 (0.80)
Day 3 (*n* = 41)	5.86 (0.73)	5.40 (0.94)
Day 14 (*n* = 39)	4.38 (1.32)	4.22 (1.00)

**Table 4:** Within-group comparison of Bristol Stool Scale scores between study visits.

Treatment Group	Visit Comparison	Difference	95% CI	*p*-Value
ORS + placebo	Day 1–Day 3	0.429	−0.216–1.07	0.2562
ORS + placebo	Day 1–Day 14	1.905	1.261–2.55	<0.0001
ORS + placebo	Day 3–Day 14	1.476	0.832–2.12	<0.0001
ORS + ABB C22^®^	Day 1–Day 3	0.625	−0.027–1.28	0.0631
ORS + ABB C22^®^	Day 1–Day 14	1.803	1.132–2.47	<0.0001
ORS + ABB C22^®^	Day 3–Day 14	1.178	0.499–1.86	0.0002

The *p*-values were obtained using a mixed-effects model for repeated measures (MMRM), adjusted for baseline values. The model includes interaction terms between treatment and visits (day 1, day 3, and day 14), allowing for the evaluation of differences within each treatment over time.

## Data Availability

The data presented in this study are available on reasonable request from the corresponding author.

## Supplementary Materials

The following supporting information can be downloaded at: https://www.mdpi.com/article/10.3390/geriatrics10020034/s1, Table S1: Serum inflammatory biomarkers (C-reactive protein) at study visits.
