# Supplementary material for: Mitigating Diarrhoea-Related Inflammation in Frail Older Adults with Postbiotic-Enhanced Oral Rehydration Solution: Insights from a Randomised, Double-Blind, Placebo-Controlled Study
Source: Geriatrics (Basel). 2025 Mar 1;10(2):34. doi: 10.3390/geriatrics10020034 (PMC11932196; doi:10.3390/geriatrics10020034)
Supplement: Supplementary file 1 [file geriatrics-10-00034-s001.zip › geriatrics-3409420-supplementary.pdf]

Supplementary material.

Table S1. Serum inflammatory biomarkers (C-reactive protein) at study visits.

**Biomarker and study visit**	**n**	**ORS + placebo n=21**	**ORS + ABB C22® n=21**	**p-value***
Serum C-reactive protein: Mean (SD) mg/L				
Day 1	40	27.1 (39.1)	12.0 (10.7)	0.525
Day 3	40	22.2 (29.6)	16.1 (16.4)	0.466
Day 14	36	30.3 (39.8)	15.1 (20.4)	0.594

C-reactive protein levels (mg/L) are shown as mean (standard deviation). * The p-values were obtained using a mixed-effects model for repeated measures (MMRM), adjusted for baseline values. The model includes interaction terms between treatment and visits (day 1, day 3, and day 14), allowing for the evaluation of differences within each treatment and between treatments over time. Serum C-reactive protein levels in the ORS + ABB C22® group stayed consistently close to baseline throughout the study. In contrast, the ORS + placebo group showed a notable decrease at day 3, followed by an increase that exceeded baseline values.
